# Laparoscopic closure of small bowel perforation: Technique of small bowel anchoring to the abdominal wall

**DOI:** 10.4103/0972-9941.55109

**Published:** 2009

**Authors:** Rajan B Jagad

**Affiliations:** Department of Surgery, New Civil Hospital and Government Medical College, Surat, Gujarat, India

**Keywords:** Bowel anchoring, laparoscopy, small bowel perforation

## Abstract

**INTRODUCTION:**

More and more complicated laparoscopic abdominal surgeries are now being performed across the world. Laparoscopic suturing of the bowel perforations is being performed by experienced surgeons. We have developed our own technique of small bowel anchoring to the abdominal wall before suturing the perforation.

**OUR MODIFICATION:**

A single stitch is taken at the corner of the perforation. The long end of the suture is retrieved by a suture retrieval needle and the small bowel is anchored to the abdominal wall. Rest of the bowel perforation is suture by the intracorporeal knot-tying technique.

**ADVANTAGES:**

Anchoring the bowel to the abdominal wall helps in fixation of the bowel to be sutured. This helps specifically for large perforation. Suturing and knot tying is relatively easy by this technique.

## INTRODUCTION

The advent of laparoscopic surgery has added a new dimension to intraabdominal surgical procedures. Almost all complicated intraabdominal surgeries can be performed by laparoscopy.[1,2] Many reports are now available regarding laparoscopic closure of gastrointestinal perforations that includes closure of typhoid perforation or sigmoid colon perforation.[3,4] Closure of the duodenal or sigmoid perforation may not create a problem, as they are fix structures. Closure of the small bowel perforation is more challenging as the small bowel is not a fix structure. During suturing and knot tying, movement of the bowel may create some difficulties, especially when the perforation is large. We have developed our own technique for the closure of large small bowel perforations.

## OUR MODIFICATION

**Figure 1:**
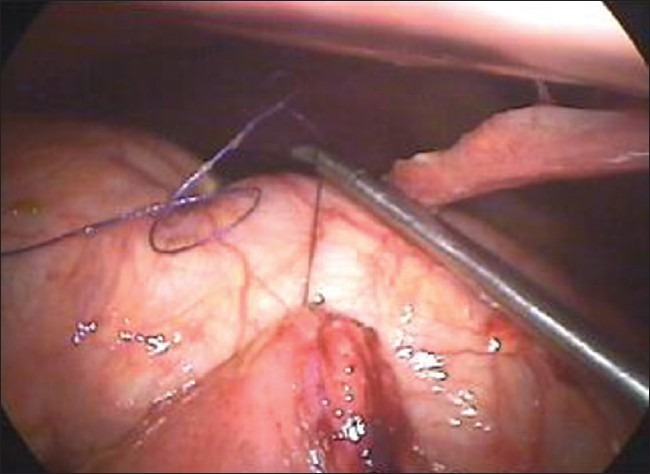
A stitch is taken at the corner of the perforation

**Figure 2:**
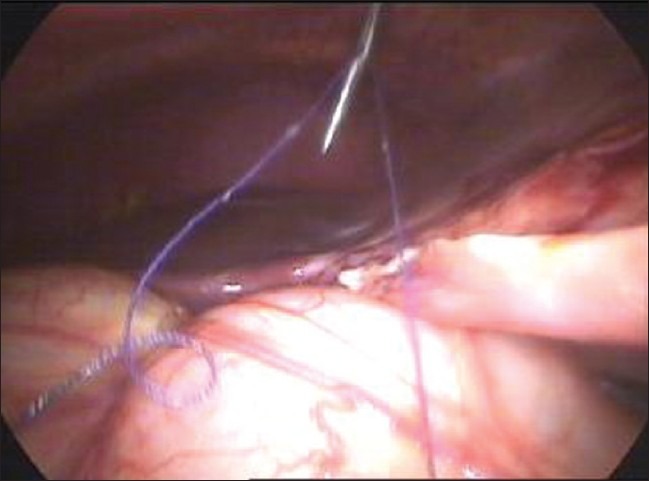
The thread is taken out of the abdomen by suture passer needle

**Figure 3:**
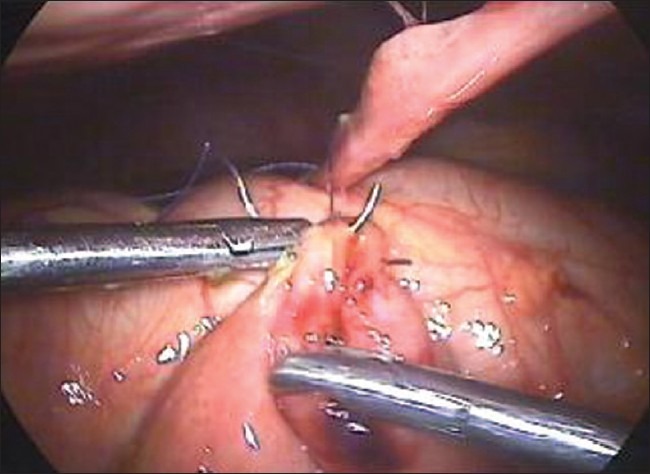
The bowel is anchored to the abdominal wall

**Figure 4:**
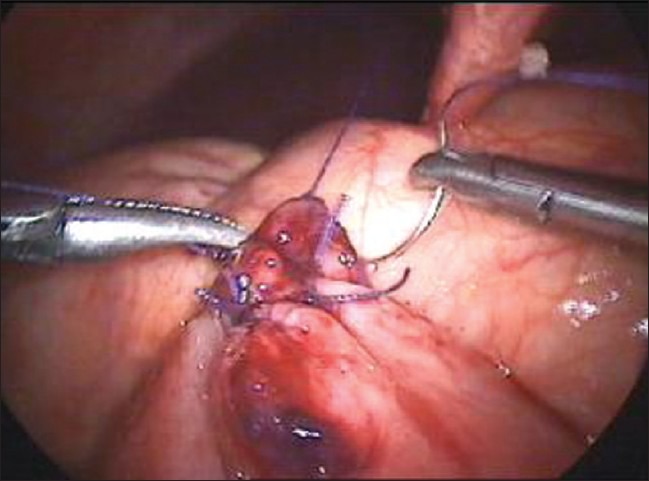
Perforation of the bowel is sutured

For the closure of large perforations of the small bowel, we adopt our technique of anchoring the small bowel with the abdominal wall. We take the single stitch at the corner of the perforation and tie the suture [Figure 1]. One end of the suture is kept long and is retrieved through the anterior abdominal wall using the suture retrieval needle [Figure 2]. We pull the suture in such a way that the bowel hangs at the middle of the abdominal wall cavity [Figure 3]. A hemostatic forceps is applied at the end of the suture that is outside the abdominal wall. Now rests of the sutures are taken [Figure 4].

## ADVANTAGES

The conventional method for laparoscopic closure of small bowel perforation is to take a simple stitch through the bowel wall. There may not be any difficulty during this maneuver if the perforation is small and two or three stitches are to be taken. The problem arises when the perforation is very large, like traumatic jejunal perforation or closure of the enterostomy after wedge resection of the Meckel's diverticulum. Because the small bowel is not a fix structure, closure of such a large perforation is difficult. By adopting our new method for anchoring the small bowel, we have observed that the suturing is easier. The small bowel is hanging in front of the camera and the perforation to be suture is just in front of the working instruments. As the bowel is hanging, it is much easier to take suture and tying a knot than the bowel that is lying free on the floor of the abdomen.
